# The role of maturation in upper-limb plyometric vs. technical plyometric training for youth badminton players

**DOI:** 10.3389/fphys.2026.1765643

**Published:** 2026-01-27

**Authors:** Yujie Shi, Mingbing Yi, Ruibao Cai, Han Li, Danni Luo, Mengjie Yu

**Affiliations:** 1 College of Physical Education, Chizhou University, Chizhou, Anhui, China; 2 College of Physical Education, Anhui Normal University, Wuhu, China

**Keywords:** badminton, injury prevention, maturation, muscle power, resistance training, sports performance

## Abstract

**Background:**

Plyometric training may enhance upper-limb explosive performance and stroke velocity in youth badminton players while contributing to mitigate injury risk, yet the influence of biological maturation on these adaptations remains unclear.

**Purpose:**

To compare the effects of upper-limb plyometric training (PLYOgen), technical plyometric training integrating badminton stroke mechanics (PLYObad), and regular training on upper-limb strength, plyometric performance, and smash speed while accounting for maturity offset.

**Methods:**

Sixty-two male players (12–14 years) were randomized to PLYOgen, PLYObad, or control. All groups continued their usual badminton practice (2–3 sessions/week; 75–90 min/session). Over 6 weeks, only the intervention groups completed an additional supervised plyometric session once per week (25–30 min; 72 explosive actions), whereas the control group performed no additional plyometric/strength sessions. Repeated-measures ANCOVA with maturity offset as covariate assessed pre–post changes in overhead medicine ball throw, seated chest pass, plyometric push-up height, and smash speed.

**Results:**

Significant time effects were observed for all outcomes (p < 0.001; η^2^p = 0.737–0.954). Time × maturity-offset interactions were significant for overhead throw (p < 0.001; η^2^p = 0.571), chest pass (p < 0.001; η^2^p = 0.482), push-up height (p = 0.006; η^2^p = 0.122), and smash speed (p < 0.001; η^2^p = 0.360), indicating that players with higher maturity offset (closer to or beyond PHV) tended to show larger pre–post improvements. Time × group interactions were also significant for overhead throw (p < 0.001; η^2^p = 0.918), chest pass (p < 0.001; η^2^p = 0.840), push-up height (p < 0.001; η^2^p = 0.718), and smash speed (p < 0.001; η^2^p = 0.950). Post-hoc analysis showed PLYOgen and PLYObad improved overhead throw and smash speed more than control, with PLYOgen also presenting greater values than control in push-up height.

**Conclusion:**

Both plyometric approaches enhanced upper-limb explosive performance, with biological maturation significantly moderating training responsiveness. This should be considered when modifying youth training programs to manage injury risk factors and to ensure that training practices are appropriately aligned with the players’ developmental level.

## 1 Introduction

Badminton is a high-intensity intermittent racket sport characterized by frequent accelerations, rapid changes of direction, repeated jumps, and powerful overhead strokes, which together impose substantial neuromuscular and coordinative demands on players (Laffaye et al., 2015; Phomsoupha and Laffaye, 2015). Match-analysis studies show that modern badminton involves thousands of strokes per match, with rallies consisting of short, explosive bouts interspersed with brief recovery periods, and shuttlecock velocities during the smash frequently exceeding 200–250 km·h^−1^, underscoring the requirement for exceptional explosive strength and technical proficiency in the upper and lower limbs (Phomsoupha and Laffaye, 2014). In youth badminton players, these demands are imposed while athletes are still developing movement competency and technical consistency, and while growth- and maturation-related changes can transiently influence motor control and postural stability. In this context, the forehand smash and jump smash are decisive attacking strokes contributing disproportionately to winning points, emphasizing the value of developing maximal (peak) stroke velocity and associated upper-limb explosive power during adolescence, when athletes are transitioning toward higher stroke speeds and more aggressive attack patterns while maintaining technical control (Laffaye et al., 2015; Edmizal et al., 2024; Towler and King, 2023). Skill-related physical fitness components such as speed, agility, power, balance, and coordination are strongly associated with badminton performance as maximal smash speed (explosive capability) and differentiating competitive levels, particularly during adolescence when players transition from basic technique acquisition to high-intensity competitive play and when on-court change-of-direction performance is already meaningfully associated with sprint and jump capabilities in youth badminton athletes (Phomsoupha and Laffaye, 2015).

Plyometric training is widely recognized as an efficient method for enhancing neuromuscular performance through exercises that exploit the stretch-shortening cycle (SSC), thereby improving the ability to generate high forces in short contraction times (Wang and Xu, 2025; Asadi et al., 2017). Systematic reviews and meta-analyses in youth athletes show that plyometric jump training (PJT) improves countermovement jump, horizontal jump, sprint speed, and change-of-direction (COD) performance, with small-to-large effect sizes across multiple team sports (Asadi et al., 2017; Ramirez-Campillo et al., 2023; Lin et al., 2025). Recent evidence further indicates that maturation stage moderates these adaptations, with generally greater improvements in jump and COD performance in pre- and post-peak height velocity (PHV) athletes compared with their mid-PHV peers (Lin et al., 2025; Silva et al., 2022; Chen et al., 2024). At the same time, upper-body plyometric training, typically implemented via medicine-ball throws, explosive push-ups, and rapid stretch-shortening actions of the shoulder girdle and elbow extensors, has been shown to enhance maximal strength, medicine-ball throwing performance, and sport-specific throwing or striking actions in youth and young adults (Wang and Xu, 2025; Garcia-Carrillo et al., 2023). However, the application of upper-limb plyometrics to racket sports remains comparatively underexplored, and the interaction between such training and technical stroke execution has seldom been investigated in adolescentes (Garcia-Carrillo et al., 2023; Li et al., 2023).

In badminton specifically, a growing body of experimental work has revealed that plyometric interventions can improve jump performance, agility, sprint speed, and overall skill-related physical fitness (Lu et al., 2022; Gepfert et al., 2025; Bozdoğan and Kızılet, 2017; Nugroho et al., 2022). A recent systematic review and meta-analysis including 11 randomized controlled trials concluded that plyometric training produces small-to-moderate improvements in power, agility, speed, and balance in badminton players, although effects on reaction time remain unclear and overall certainty of evidence is low to very low (Deng et al., 2024). Narrative and scoping reviews have similarly highlighted the potential of plyometric drills such as jumps, bounds, and jump smashes to enhance agility, court movement, and explosive stroke production in badminton, while also contributing to injury-prevention by strengthening muscles and connective tissues (Shedge et al., 2024).

Nevertheless, most badminton-specific plyometric programs have emphasized lower-limb actions (e.g., vertical jumps, drop jumps, multi-directional hops), with limited emphasis on structured upper-limb plyometric exercises designed to improve racket-arm power and shuttlecock velocity (Deng et al., 2024; Shedge et al., 2024). Moreover, although some interventions have incorporated sport-specific elements such as jump smash repetitions and multi-shuttle drills, these have rarely been conceptualized or evaluated as “technical plyometric training” that explicitly integrates plyometric loading with stroke mechanics (Shedge et al., 2024; Chou, 2022). This distinction is meaningful because smash performance is determined not only by local upper-limb power, but also by whole-body kinetic-chain coordination and segmental positioning at impact that influence racket/shuttlecock speed (Phomsoupha and Laffaye, 2015). Accordingly, embedding plyometric loading within the stroke (technical plyometrics) is expected to enhance the expression of rapid force production under sport-specific joint angles, contraction velocities, and intermuscular timing which are factors known to influence transfer from strength/power training to skilled performance (Young, 2006). In contrast, generic upper-limb plyometrics (for instance medicine-ball throws, explosive push-ups) can increase general upper-body power and throwing/propulsion performance, yet improvements in non-specific tasks do not always translate proportionally to sport-specific high-speed skills when coordination and movement constraints differ (Turgut et al., 2019).

Biological maturation is a determinant of training responsiveness during adolescence, with peak height velocity (PHV) representing a critical milestone for neuromuscular development, growth in lean mass, and changes in muscle–tendon architecture (Mirwald et al., 2002; Tsutsui et al., 2022). Maturation status is typically indexed using age at PHV or maturity offset equations, which capture individual variation in the timing of the adolescent growth spurt and are recommended over chronological age for structuring long-term athlete development (Mirwald et al., 2002). Studies in youth athletes indicate that strength and power development are substantially influenced by maturation, with early-maturing individuals often outperforming their later-maturing peers, but with transient decrements in motor coordination and performance around PHV (“adolescent awkwardness”) (Gryko et al., 2022; Retzepis et al., 2025; Meylan et al., 2014).

Meta-analyses focusing on plyometric jump training have shown that pre- and post-PHV athletes typically achieve meaningful gains in jump and sprint performance, whereas adaptations during mid-PHV are often smaller on average, possibly due to transient disruptions in posture control and neuromuscular coordination reported around the period of rapid growth (Ramirez-Campillo et al., 2023; Lin et al., 2025; Chen et al., 2024). Although some long-term athlete development frameworks have described discrete “windows of opportunity,” the existence of generic, ability-independent windows is debated and the empirical support is considered limited, thus maturation is better treated as a continuous moderator of trainability rather than as fixed chronological-age windows (Ford et al., 2011; Varghese et al., 2022). Importantly, the age range of 12–14 years is sensitive because it straddles the average timing of peak height velocity (PHV) in boys and, given the well-documented between-athlete variability in PHV timing, naturally includes athletes who are meaningfully pre-, circa-, and post-PHV within the same chronological band (Mirwald et al., 2002). This maturational heterogeneity is particularly relevant for plyometric interventions because the rapid growth phase around PHV is associated with measurable changes in neuromuscular control and, in some cohorts, heightened inter-limb asymmetries during jump/landing tasks, which can plausibly influence both tolerance and responsiveness to stretch–shortening-cycle loading (Read et al., 2018). In parallel, maturation across pre-, circa-, and post-PHV is associated with changes in musculoskeletal morphology, which may modify force-production characteristics and therefore the magnitude of adaptation to explosive training stimuli (Radnor et al., 2020).

Despite the rapidly expanding literature on plyometric training and maturation, several important gaps remain in relation to badminton and upper-limb performance. The vast majority of maturation-specific plyometric research has been conducted in team sports such as soccer and basketball and has focused on lower-limb outcomes (e.g., countermovement jump, sprint, COD), with little attention to upper-limb explosive strength or sport-specific striking performance (Asadi et al., 2017; Ramirez-Campillo et al., 2023; Lin et al., 2025; Chen et al., 2024). Moreover, although upper-body plyometric training has been shown to improve medicine-ball throwing and sport-specific overhead skills in other sports, there is a scarcity of randomized controlled trials examining its effects on racket-sport strokes such as the badminton smash, particularly in youth (Garcia-Carrillo et al., 2023; Syafii et al., 2024; Kamaruddin and Indah, 2025). Importantly, badminton should not be viewed in isolation since overhead throwing/serving/spiking actions in baseball, tennis, volleyball, and handball share biomechanical similarities with the badminton smash, including proximal-to-distal sequencing and kinetic-chain energy transfer from the trunk and lower limbs to the upper extremity (Kovacs and Ellenbecker, 2011; Reeser et al., 2010; Seroyer et al., 2010). Consistent with this biomechanical overlap, upper-extremity plyometric training has been reported to improve throwing velocity in baseball and serve-related velocity outcomes in tennis, and systematic reviews conclude that upper-body plyometrics meaningfully improve medicine-ball throw performance and sport-specific overhead performance across populations (Garcia-Carrillo et al., 2023; Carter et al., 2007; Deng et al., 2022). Additionally, existing badminton-specific plyometric interventions in youth players have primarily targeted lower-limb power and agility, without directly comparing generic upper-limb plyometric programs to sport-specific technical plyometric protocols that integrate jump smash or attack-stroke mechanics (Deng et al., 2024). Also, even though recent syntheses have emphasized the importance of reporting maturity status, most badminton studies in adolescents have either ignored maturation or relied solely on chronological age, thereby masking the potentially distinct adaptations of pre-, mid-, and post-PHV players within a narrow age band such as 13–14 years (Ramirez-Campillo et al., 2023; Chen et al., 2024; Deng et al., 2024). Finally, there is a paucity of research simultaneously assessing both physical outcomes (e.g., upper-limb strength and power) and badminton-specific technical performance (e.g., attack stroke or smash speed) when contrasting different plyometric modalities in youth players at varying maturation stages (Phomsoupha and Laffaye, 2014; Gepfert et al., 2025; Deng et al., 2024). This gap is relevant because smash speed is a performance-discriminative endpoint and is coupled to racket-head speed and whole-body kinetic-chain sequencing during the jump smash (Phomsoupha and Laffaye, 2014; Ramasamy et al., 2024).

Therefore, the present study aims to compare the effects of upper-limb plyometric training, technical plyometric training integrating badminton-specific stroke mechanics, and regular badminton training (control) on upper-limb strength and power and maximum velocity attack stroke (smash) in youth badminton players. By classifying participants according to biological maturation status within 13–14 age range, the study further seeks to determine whether the effectiveness of upper-limb versus technical plyometric training differs across maturation stages. It is hypothesized that both plyometric interventions will elicit greater improvements in upper-limb physical performance and attack stroke speed than the control condition, and that technical plyometric training will confer superior gains in badminton-specific attack performance due to its closer biomechanical specificity, with the magnitude of these adaptations being moderated by maturation status (Ramirez-Campillo et al., 2023; Li et al., 2023; Gepfert et al., 2025).

## 2 Methods

### 2.1 Study design

The present investigation was designed as a parallel-group, three-arm, randomized, controlled experiment evaluating the effects of two distinct upper-limb plyometric training modalities (generic upper-limb plyometric exercise [PLYOgen] and badminton-specific technical plyometric training [PLYObad] compared with a control condition with no plyometric intervention) in youth badminton players. Participants were randomly allocated in a 1:1:1 ratio to one of the three intervention groups after baseline testing, using concealed, computer-generated randomization stratified by biological maturation status to ensure balanced distribution of PHV individuals across groups. The randomization sequence was generated by an independent researcher who had no role in participant recruitment, baseline testing, or outcome assessment. Participant enrolment was conducted by other researcher, who confirmed eligibility and obtained consent/assent. Group assignment was performed using the pre-generated allocation schedule, with allocation concealment maintained via that was accessed only after completion of baseline testing. Neither athletes, coaches, nor assessors from the participating clubs were directly involved in the design of the intervention protocols, the selection of outcomes, or the reporting of the study. However, public involvement was incorporated pragmatically, as the training schedules, feasibility of session duration, and acceptability of exercises were discussed with club coaches and youth coordinators to ensure safe integration of the intervention into the existing training calendar.

The study was implemented over a 6-week period, with evaluations conducted immediately before and after the intervention. All training sessions and testing procedures were delivered at a single site within the same country to ensure logistical consistency. The protocol prespecified all primary and secondary outcomes, including upper-limb explosive strength, upper-limb plyometric performance, and badminton attack-stroke velocity. No outcome measures or analyses were added or modified after trial commencement. No changes to the intervention structure, frequency, or content were required once the trial had begun, and no deviations from the original methodology occurred that would affect internal validity.

### 2.2 Participants

Participants were recruited from regional badminton training centers and competitive youth development programs through direct contact with club directors, informational sessions with athletes and parents, and distribution of study invitations during scheduled training hours. Eligibility criteria required that all participants be male adolescent badminton players aged 12–14 years, engaged in structured badminton training for a minimum of 2 years, free from musculoskeletal injury in the preceding 3 months, and medically cleared training activities. Additional inclusion criteria specified participation in at least two organized badminton sessions per week and absence of any medical or developmental conditions that could influence growth, maturation, or neuromuscular performance. Players undergoing medical treatment, presenting chronic pain, or participating in external strength and conditioning programs outside their club regimen were excluded to avoid confounding training loads. After eligibility screening and baseline anthropometric and maturity assessments, athletes and their legal guardians provided written informed consent, and participants were randomized into one of the three study groups.

**FIGURE 1:**
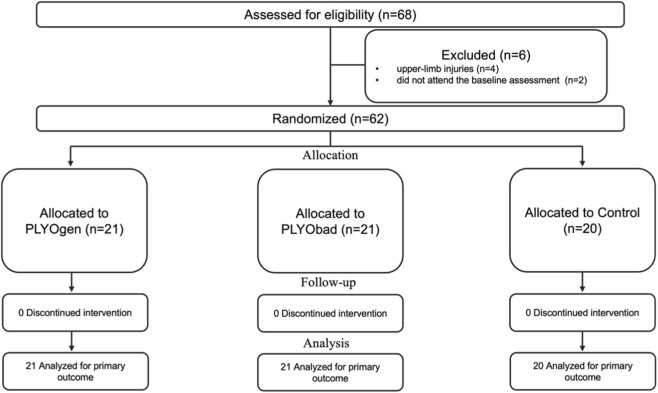
Participants allocation. PLYOgen: Upper-Limb Plyometric Training; PLYObad: Technical Plyometric Training.

From an initial pool of 68 volunteers, only 62 were included (Figure 1). Four had upper-limb injuries and two did not attend the baseline assessment. Overall, the 62 participants were 12.8 ± 0.6 years old, with an average height of 151.4 ± 10.2 cm and sitting height of 78.0 ± 5.1 cm. Their mean body mass was 44.2 ± 8.6 kg, corresponding to an average BMI of 19.1 ± 2.0 kg/m^2^. In terms of maturity, the sample was on average 1.4 ± 0.8 years before PHV, with a mean age at PHV of 14.2 ± 0.5 years. The players were randomly allocated to the PLYOgen (n = 21), PLYObad (n = 21), or control (n = 20) groups. Table 1 summarizes the demographic and physical characteristics of the participants.

**TABLE 1:** Characteristics of participants.

Outcome	PLYOgen	PLYObad	Control
N	21	21	20
Age (years)	12.8 ± 0.6	12.9 ± 0.5	12.8 ± 0.5
Height (cm)	150.5 ± 10.8	151.7 ± 9.5	151.9 ± 10.4
Sitting height (cm)	77.6 ± 5.7	78.3 ± 5.0	78.1 ± 4.8
Body mass (kg)	42.6 ± 7.6	44.7 ± 9.1	45.2 ± 9.1
BMI (kg/m^2^)	18.7 ± 1.7	19.2 ± 2.1	19.4 ± 2.1
Maturity offset (years)	−1.4 ± 0.9	−1.3 ± 0.8	−1.3 ± 0.8
Age at PHV (years)	14.2 ± 0.6	14.2 ± 0.5	14.1 ± 0.5

BMI: body mass index; PLYOgen: Upper-Limb Plyometric Training; PLYObad: Technical Plyometric Training.

All eligible players who consented were assigned to a group after baseline testing, and all participants were asked to complete the 6-week intervention and post-testing following identical schedules. Training adherence was monitored through attendance logs maintained by the research staff in collaboration with team coaches, documenting presence, session completion, and any deviations from the prescribed program. Athletes were instructed to maintain their usual badminton practice routines outside the intervention sessions but refrain from initiating new strength or conditioning activities during the study period. Across the intervention window, adherence remained high, with the majority of enrolled participants completing at least 90% of the prescribed training sessions, and reasons for occasional absence included academic commitments, or temporary family obligations. No adverse events or training-related injuries were reported that required withdrawal. All study procedures were reviewed and approved by the institutional ethics committee [ANHUI NORMAL UNIVERSITY, AHNU-ET2025069], conducted in accordance with the Declaration of Helsinki, and designed to ensure the safety, confidentiality, and wellbeing of all participating youth athletes.

### 2.3 Sample size

A moderate interaction effect size of Cohen’s f = 0.25 was selected for the group × time term, compatible with the pooled standardized mean differences for medicine ball throw and sport-specific throwing performance reported in a meta-analysis (Garcia-Carrillo et al., 2023). Using a repeated-measures ANOVA approximation with three groups, two measurements (pre, post), α = 0.05, desired power (1 − β) = 0.80, and an assumed correlation of 0.70 between repeated measures, standard power calculations indicate a required total sample size of approximately 60 participants to detect this moderate group × time interaction.

### 2.4 Training interventions

The intervention consisted of two experimental plyometric training programmes and one control condition, all integrated into the players’ regular technical–tactical badminton training over a 6-week period. All groups continued their usual badminton practice (two to three sessions per week, 75–90 min per session), and only the experimental groups performed an additional, supervised upper-limb–focused plyometric session once per week. A once-weekly frequency was selected as a pragmatic, feasibility-driven dose intended to maximize adherence within youth schedules and to minimize disruption or interference with technical–tactical training, while limiting the incremental weekly workload imposed by the intervention. The two plyometric programmes were carefully matched for weekly training volume and structure to isolate the effect of exercise content. Although meta-analyses of plyometric jump training in youth commonly report effective programmes implemented 2–3 sessions per week over 6–8 weeks (Slimani et al., 2016; Asadi et al., 2016), available evidence from volume-equated comparisons indicates that one session per week can still produce meaningful adaptations in youth athletes, particularly when integrated into ongoing sport practice (Bianchi et al., 2019; Clemente et al., 2025).

Each plyometric session lasted approximately 25–30 min, took place immediately after a standardized dynamic warm-up, and included four exercises performed in three sets of six maximal repetitions, yielding a total of 72 explosive upper-limb actions per session. This overall dose and contact number were chosen in line with previous upper-body plyometric programmes in youth racket and overhead sports (Garcia-Carrillo et al., 2023).

In the PLYOgen, the exercises consisted of generic, medicine-ball–based plyometric drills designed to target explosive pushing, overhead extension and trunk-rotation power without involving the racket. Each weekly session included four exercises performed exactly as described in table 2, namely, a seated or tall-kneeling medicine ball chest pass with a 2-kg ball, a tall-kneeling two-handed overhead throw, a standing two-handed overhead slam to the floor, and a standing rotational throw toward the dominant side. Each exercise was carried out for three sets of six maximal repetitions, totalling eighteen explosive actions per drill and seventy-two explosive upper-limb actions per session. Rest intervals were standardized with 3 minutes between sets and approximately 20 seconds between individual repetitions. Progression across the 6 weeks focused on improving execution quality and maximal intent while maintaining constant volume, with technical feedback emphasizing rapid stretch–shortening of the shoulder and elbow extensors, coordinated trunk contribution and stable lower-body bracing.

**TABLE 2:** Description of the training interventions.

Group	Exercise/Drill	Description	Sets	Reps	Explosive actions per exercise	Total explosive actions per session	Week 1	Week 2	Week 3	Week 4	Week 5	Week 6
Upper-limb plyometric training (PLYOgen)	Medicine ball chest pass (2 kg)	Seated/tall-kneeling bilateral chest pass	3	6	18	72	Yes	Yes	Yes	Yes	Yes	Yes
	Overhead throw (2 kg)	Tall-kneeling two-handed overhead throw	3	6	18		Yes	Yes	Yes	Yes	Yes	Yes
	Overhead slam (2 kg)	Standing overhead slam to the floor	3	6	18		Yes	Yes	Yes	Yes	Yes	Yes
	Rotational throw (2 kg)	Standing trunk-rotation throw	3	6	18		Yes	Yes	Yes	Yes	Yes	Yes
Technical plyometric training (PLYObad)	Stationary jump smash	Coach-fed clear; vertical jump smash	3	6	18	72	Yes	Yes	Yes	Yes	Yes	Yes
	Diagonal approach jump smash	Footwork + approach + jump smash	3	6	18		Yes	Yes	Yes	Yes	Yes	Yes
	Depth-reactive jump smash	Step off 20–30 cm box → jump smash	3	6	18		Yes	Yes	Yes	Yes	Yes	Yes
	Fast attack clear	Maximal-speed overhead attack clear	3	6	18		Yes	Yes	Yes	Yes	Yes	Yes
Control group (CON)	Regular badminton training	Habitual technical/tactical training (no plyometrics)	0	0	0	0	Yes	Yes	Yes	Yes	Yes	Yes

In the PLYObad, the same total weekly explosive volume, set–rep structure and rest intervals were applied, but each repetition was executed as a badminton-specific overhead stroke to create a technical plyometric stimulus combining stretch–shortening loading with sport-specific movements. Following the structure presented in the table 2, each session consisted of four drills, in specific, a stationary jump smash performed after a coach-fed high clear, a diagonal approach jump smash incorporating a short footwork sequence, a depth-reactive jump smash initiated by stepping off a 20 cm box into a rapid approach, and fast attack clears executed with maximal racquet-head speed. All drills were performed for three sets of six maximal strokes, matching the seventy-two-action weekly structure of the upper-limb plyometric group. Players used their own rackets and standardized shuttles, and were provided continuous coaching to reinforce optimal kinetic-chain sequencing, elevation timing, contact-point positioning and follow-through mechanics.

The control group continued its usual badminton-specific technical, tactical and conditioning training throughout the 6-week period and did not perform any additional structured plyometric or strength training. Coaches were asked to avoid introducing new jump- or throw-intensive drills during the study period beyond their habitual practice content.

### 2.5 Anthropometric assessments

Biological maturation was assessed using the maturity-offset method proposed by Mirwald and colleagues, which estimates the number of years an individual is before or after peak height velocity (PHV) based on sex-specific anthropometric predictors (standing height, sitting height, leg length, chronological age, and body mass). This non-invasive predictive equation has been widely validated and is considered an appropriate method for estimating biological maturation status in different contexts and in adolescent athletes, showing strong correlations with longitudinal PHV-derived measures and acceptable standard errors for group-level classification (Mirwald et al., 2002). Nevertheless, maturity-offset predictions include non-trivial individual-level error (commonly reported on the order of ±0.5–0.6 years), and accuracy is typically reduced when classifying individuals close to PHV (Pichardo et al., 2019). Additionally, longitudinal evaluations indicate systematic bias such that predicted timing of PHV may be overestimated in earlier-maturing youth and underestimated in later-maturing youth, which can compress inter-individual variability and misclassify maturation status at the individual level (Sullivan et al., 2023). These aspects represent limitations arising from the trade-off between real-world applicability and in-context assessments, as opposed to laboratory-based contexts.

Standing height and sitting height were measured to the nearest 0.1 cm using a wall-mounted stadiometer (SECA 213), and body mass was measured to the nearest 0.1 kg with a calibrated digital scale (SECA 876). Leg length was calculated as standing height minus sitting height. All measurements were taken with participants barefoot and wearing light clothing, following standardized anthropometric guidelines. Maturity offset was then computed using the published sex-specific regression equation (23), yielding an estimate of each athlete’s relative maturity in years from PHV (negative values indicating pre-PHV, positive values indicating post-PHV). All anthropometric assessments were conducted by the same experienced researcher to minimize inter-observer measurement error, and measurements were repeated twice, with a third measurement taken if discrepancies exceeded 0.4 cm for heights or 0.2 kg for mass.

### 2.6 Performance assessments

All evaluations were conducted in a controlled indoor sports laboratory to ensure optimal standardization of testing conditions. The evaluations were part of the athletes’ regular testing battery. Therefore, familiarity with the specific test procedures was already ensured. The testing room maintained a stable environmental profile across all sessions, with temperature held between 22 °C and 24 °C and relative humidity between 50% and 60%, values selected to minimize thermal or humidity-related variations. All assessments were scheduled between 5 and 7 p.m. to reduce circadian fluctuations. Before each testing session, participants completed a standardized warm-up consisting of 5 min of light jogging, followed by 10 min of dynamic mobility exercises targeting both upper and lower limbs, and concluding with sport-specific activation drills including submaximal jumps, accelerations, and controlled overhead shadow swings. After the warm-up, players rested for 3 min before beginning the test battery. The sequence of testing was standardized to minimize fatigue interactions, starting with the seated 3 kg medicine ball chest pass, followed by the 3 kg overhead medicine ball throw, then the plyometric push-up test on the validated contact mat, and finally the badminton smash/attack speed assessment using the Bushnell radar gun. The entire testing battery was implemented identically at baseline (pre-intervention) and after the 6-week training period (post-intervention), with all players assessed individually under the same environmental and procedural conditions to ensure comparability of performance outcomes across time.

#### 2.6.1 Seated medicine ball chest pass

The seated medicine ball chest pass consists of participants sitting with their back against a wall (to limit trunk motion), holding a 3 kg medicine ball at chest level with both hands, and, on command, explosively pushing the ball forward for maximum distance. Prior research indicates that the seated chest medicine ball throw exhibits good test–retest reliability in children and adolescents (ICC = 0.84; 95% CI [0.48–0.96]) in a meta-analysis (Varela et al., 2025). Another study in volleyball athletes reported ICC = 0.996 (p < 0.01) for a backward-overhead version of the throw, supporting very high reliability of medicine ball throws for explosive power (Stockbrugger and Haennel, 2001). A measuring tape was laid out along the floor beginning at the wall, and the distance from the release line to the first landing point of the ball was recorded in centimetres. Participants performed one familiarisation trial and then three maximal attempts, each separated by 90 s of rest. The best (longest) distance (measured in m) and the mean of the three distances were recorded as outcomes. A scale mat was placed on the floor, and the researcher used a scale to measure the exact point at which the ball dropped.

#### 2.6.2 Overhead medicine ball throw

The overhead medicine ball throw is designed to assess upper-body and trunk explosive power via an overhead (or backward-overhead) throw of a medicine ball. In our protocol, participants kneel tall (to reduce lower-body contribution), hold a 3 kg medicine ball overhead with both hands, and then explosively throw it forward for maximal distance. Literature supports good reliability for overhead medicine ball throw tests: for instance, in a study of athletes the backward overhead medicine ball throw achieved ICC = 0.86, though a learning effect was noted across trials (Mayhew et al., 2005). A measuring tape measured the distance from the release line to the first landing point of the ball. After a single familiarisation throw, participants completed three maximal attempts with 90 s of rest between them. The best distance (cm) were recorded.

#### 2.6.3 Plyometric push-up height

The plyometric push-up test evaluates upper-body stretch-shortening cycle (SSC) performance by requiring participants to push off explosively such that both hands leave the ground. Evidence indicates that upper-body plyometric push-up tests show moderate to very high reliability with ICCs ranging from 0.80 to 0.96 and coefficient of variation (CV) from 4.2% to 7.6 for variables including flight time, peak force and impulse (Hogarth et al., 2013). In our protocol, participants assumed a standard push-up position with their hands on a validated contact mat system (ChronoJump, Spain) (Stanton et al., 2019) which recorded flight time. From flight time, jump height was estimated. After instruction and one familiarisation attempt, participants performed three maximal plyometric push-ups separated by 90 s of rest. The highest jump height (in cm) was recorded as the main outcome.

#### 2.6.4 Badminton smash speed

The badminton smash and attack speed test evaluates sport-specific upper-limb and whole-body explosive performance by quantifying the peak shuttlecock velocity during a maximal forehand jump smash. Shuttlecock speed was recorded using a Bushnell Doppler radar gun, positioned behind the athlete on the racket-arm side and aligned with the shuttle trajectory according to previous procedures for projectile velocity assessment in racket sports (Makar et al., 2024). Because Doppler radar guns quantify the velocity component along the radar line-of-sight, any misalignment between the beam and the shuttle’s initial flight path can introduce systematic underestimation (i.e., cosine error), and this error increases as the angular offset increases (Smi et al., 2024). To minimize cosine error, players executed the jump smash from a standardized hitting zone and were instructed to direct all smashes toward a predefined target area within the opposite court, thereby constraining inter-trial variation in shuttle direction (Smi et al., 2024). The radar gun was mounted on a tripod and its position/tilt were standardized so that the radar line-of-sight approximated the intended initial shuttle trajectory (i.e., aligned with the line connecting the hitting zone and the target area), and alignment was verified before each athlete’s trials (Smi et al., 2024). Specifically, the Bushnell radar showed ICC = 0.989 (95% CI: 0.986–0.991) for throwing and ICC = 0.986 (95% CI: 0.983–0.989) for kicking when compared simultaneously with the Stalker radar, which served as the reference criterion; correlations with the Stalker were r = 0.988 and r = 0.973, and coefficients of variation were 1.25% and 1.18% for throwing and kicking respectively, indicating highly consistent measurement (Makar et al., 2024). Each player performed one familiarization trial and then five maximal jump-smash attempts separated by 30 s. Trials with obvious directional deviation outside the standardized target corridor were repeated to maintain a consistent shuttle trajectory relative to the radar beam (Smi et al., 2024). The peak shuttlecock velocity (km·h^−1^) served as the primary outcome.

### 2.7 Statistical procedures

All statistical analyses were performed using a repeated-measures ANCOVA to evaluate the effects of the three intervention conditions (PLYOgen, PLYObad and control) on pre-to post-intervention changes in the outcomes. The within-subject factor was Time (pre vs. post), the between-subject factor was Group (three levels), and biological maturation was incorporated as a continuous covariate using each participant’s maturity offset value (years from peak height velocity). This model allowed adjustment for inter-individual differences in maturation status.

Prior to analysis, all variables were screened for normality using Shapiro–Wilk tests, and for homogeneity of variances and covariances using Levene’s and Box’s M tests, respectively. Linearity between the covariate (maturity offset) and the dependent variables was verified, along with homogeneity of regression slopes to ensure the appropriateness of including the covariate. Outliers and influential cases were explored through standardized residuals, Cook’s distance, and leverage statistics, and no data points exceeded accepted influence thresholds.

For each outcome, the repeated-measures ANCOVA estimated the adjusted means at each time point and the adjusted mean change scores. Significant interaction effects were followed by Bonferroni-corrected pairwise comparisons to identify between-group differences in adjusted pre–post changes. Partial eta squared (η^2^p) was reported as the measure of effect size for main and interaction terms, interpreted using conventional benchmarks (0.01 small, 0.06 moderate, 0.14 large). For all analyses, statistical significance was set at p < 0.05. To complement frequentist inference, 95% confidence intervals were provided for adjusted mean differences and effect sizes. All statistical analyses were conducted in SPSS (version 27, IBM Corp., Armonk, NY).

## 3 Results

**FIGURE 2:**
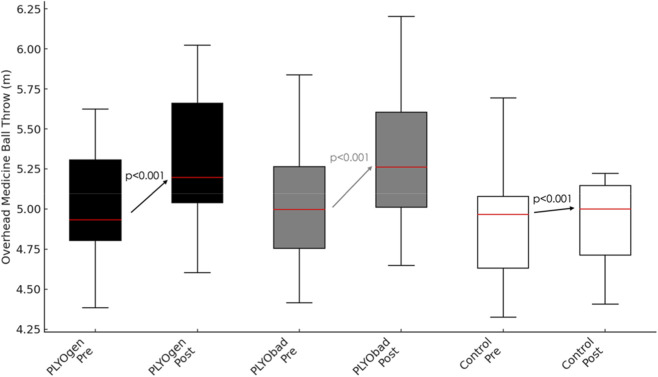
Pre–post changes in Overhead Medicine Ball Throw performance (m) for PLYOgen, PLYObad, and Control groups. PLYOgen: Upper-Limb Plyometric Training; PLYObad: Technical Plyometric Training.

Considering the overhead medicine ball throw there was a very large main effect of time (p < 0.001, η^2^p = 0.954). This change over time was strongly moderated by maturity offset (Time × Maturityoffset interaction, p < 0.001, η^2^p = 0.571), and by group (Time × group interaction, p < 0.001, η^2^p = 0.918). Between subjects, the covariate maturity offset had a significant effect on overall overhead medicine ball throw performance (p < 0.001, η^2^p = 0.394) indicating that more biologically mature participants displayed greater throwing distances when averaged across time and groups. After controlling for maturity offset, the main effect of group on the time-averaged overhead throw was not statistically significant (p = 0.065, η^2^p = 0.090). Comparison between the two plyometric interventions showed no statistically significant difference in adjusted pre–post change (PLYOgen vs. PLYObad: Δ = 0.006 m; 95% CI −0.013 to 0.025; p = 0.526). The Time × MaturityOffset interaction reflected a positive directionality, such that athletes with higher maturity offset values (closer to/after PHV) exhibited larger improvements; each +1.0 years in maturity offset was associated with an additional +0.046 m improvement (95% CI 0.035 to 0.056; p < 0.001). Comparisons between groups and *post hoc* results are presented in Table 3 and Figure 2 shows the pre–post comparisons for each group.

**TABLE 3:** Descriptive statistics and between-group comparisons for each time point.

Outcome	Moment	PLYOgen (n = 21)	PLYObad (n = 21)	Control (n = 20)	Group comparisons per moment	Significant *post hoc* comparisons
Overhead medicine ball throw (m)	Pre	4.99 ± 0.37	5.06 ± 0.40	4.94 ± 0.39	*p* = 0.542, η^2^p = 0.021	
	Post	5.29 ± 0.41	5.36 ± 0.45	5.01 ± 0.40	*p* = 0.003, η^2^p = 0.185	PLYOgen > control (p = 0.010); PLYObad > control (p = 0.006)
Seated medicine ball chest pass (m)	Pre	3.99 ± 0.34	4.01 ± 0.30	4.02 ± 0.32	*p* = 0.973, η^2^p = 0.001	
	Post	4.14 ± 0.36	4.16 ± 0.32	4.06 ± 0.34	*p* = 0.417, η^2^p = 0.030	
Plyometric push-up height (cm)	Pre	2.78 ± 0.35	2.82 ± 0.33	2.81 ± 0.35	*p* = 0.996, η^2^p < 0.001	
	Post	3.10 ± 0.31	3.07 ± 0.37	2.84 ± 0.37	*p* = 0.010, η^2^p = 0.148	PLYOgen > control (p = 0.011)
Badminton smash speed (km/h)	Pre	123.23 ± 8.49	128.12 ± 11.99	124.05 ± 11.96	*p* = 0.368, η^2^p = 0.034	
	Post	135.89 ± 9.43	141.12 ± 13.40	125.73 ± 12.62	*p <* 0.001, η^2^p = 0.262	PLYOgen > control (p = 0.007); PLYObad > control (p < 0.001)

PLYOgen: Upper-Limb Plyometric Training; PLYObad: Technical Plyometric Training.

**FIGURE 3:**
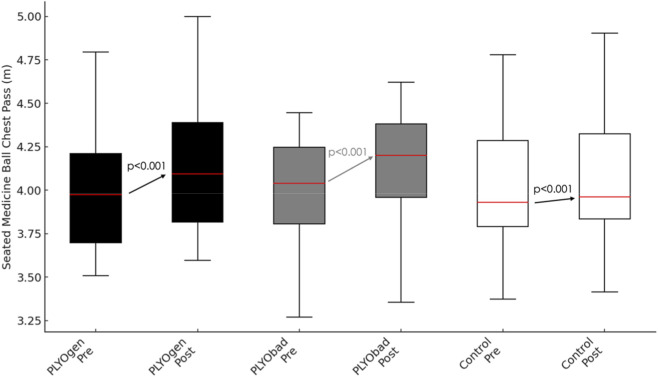
Pre–post changes in Seated Medicine Ball Chest Pass performance (m) for PLYOgen, PLYObad, and Control groups. PLYOgen: Upper-Limb Plyometric Training; PLYObad: Technical Plyometric Training.

There was a very large main effect of time on Seated Medicine Ball Chest Pass (p < 0.001, η^2^p = 0.924). This temporal change was significantly moderated by maturity offset (Time × Maturityoffset interaction, p < 0.001, η^2^p = 0.482), and by group (Time × group interaction, p < 0.001, η^2^p = 0.840). In the between-subjects tests, maturity offset had a significant effect on overall seated chest pass performance when averaged across time and groups (p < 0.001, η^2^p = 0.348). After controlling for maturity offset, the main effect of group on the time-averaged chest pass distance was not statistically significant (p = 0.798, η^2^p = 0.008). Comparison of the two plyometric interventions did not show a significant difference in adjusted pre–post change (PLYOgen vs. PLYObad: Δ = 0.009 m; 95% CI −0.004 to 0.021; p = 0.169). The Time × MaturityOffset interaction was also positive in direction since greater maturity offset predicted larger improvements (+0.026 m per +1.0 years; 95% CI 0.019 to 0.033; p < 0.001). Comparisons between groups and *post hoc* results are presented in Table 3 and Figure 3 shows the pre–post comparisons for each group.

**FIGURE 4:**
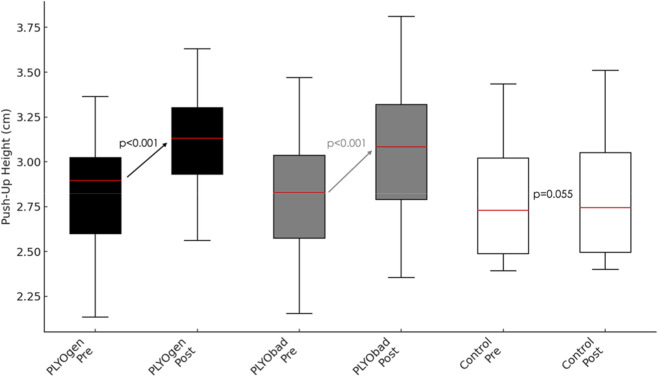
Pre–post changes in Push-Up Flight Height (cm) for PLYOgen, PLYObad, and Control groups. PLYOgen: Upper-Limb Plyometric Training; PLYObad: Technical Plyometric Training.

There was a large main effect of time on push-up height (p < 0.001, η^2^p = 0.737) (Figure 4). This change over time was significantly moderated by maturity offset (Time × Maturityoffset interaction, p = 0.006, η^2^p = 0.122), and by group (Time × group interaction, p < 0.001, η^2^p = 0.718). Between subjects, maturity offset had a significant effect on overall plyometric push-up height when averaged across time and groups (p < 0.001, η^2^p = 0.284). After adjusting for maturity offset, the main effect of group on the time-averaged plyometric push-up height was not statistically significant (p = 0.319, η^2^p = 0.039). PLYOgen showed a greater adjusted improvement than PLYObad (PLYOgen vs. PLYObad: Δ = 0.065 cm; 95% CI 0.018 to 0.112; p = 0.008). Directionality of the Time × MaturityOffset interaction indicated larger improvements in more mature athletes (+0.036 cm per +1.0 years; 95% CI 0.011 to 0.061; p = 0.006).

**FIGURE 5:**
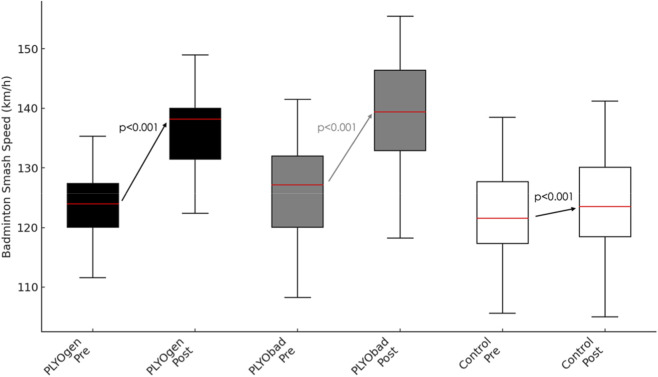
Pre–post changes in Badminton Smash Speed (km/h) for PLYOgen, PLYObad, and Control groups. PLYOgen: Upper-Limb Plyometric Training; PLYObad: Technical Plyometric Training.

There was a very large main effect of time on smash speed (p < 0.001, η^2^p = 0.953) (Figure 5). This improvement over time was significantly moderated by maturity offset (Time × Maturityoffset interaction, p < 0.001, η^2^p = 0.360), and by group (Time × group interaction, p < 0.001, η^2^p = 0.950). In the between-subjects analyses, maturity offset had a significant effect on overall badminton smash speed when averaged across time and groups (p = 0.001, η^2^p = 0.165). After adjusting for maturity offset, there was also a significant main effect of group on the time-averaged smash speed, (p = 0.022, η^2^p = 0.123). The two plyometric interventions did not differ significantly in adjusted smash-speed improvements (PLYOgen vs. PLYObad: Δ = 0.10 km·h^−1^; 95% CI −0.57 to 0.76; p = 0.775). The Time × MaturityOffset interaction was positive since each +1.0 years in maturity offset was associated with an additional +1.13 km·h^−1^ increase in smash speed (95% CI 0.73 to 1.53; p < 0.001), indicating that athletes closer to/after PHV tended to be more responsive.

To test whether maturation moderated the between-intervention contrast (generic vs. technical), we examined the Group × MaturityOffset interaction on pre–post change (equivalent to a Time × Group × MaturityOffset moderation test). No significant moderation of the PLYOgen–PLYObad contrast was detected for overhead throw (p = 0.288), chest pass (p = 0.998), push-up height (p = 0.972), or smash speed (p = 0.093), suggesting that maturity primarily influenced the magnitude of improvement similarly across interventions rather than selectively favoring one intervention.

## 4 Discussion

The present study revealed that both plyometric groups (PLYOgen and PLYObad) presented substantial improvements over time in overhead medicine ball throw, seated medicine ball chest pass, plyometric push-up height, and badminton smash speed in youth participants, and that biological maturity (maturity offset) significantly moderated most of these training adaptations. The covariate maturity offset showed significant interactions with time (e.g., overhead throw, smash speed), indicating that more mature participants tended to show greater adaptation. Post hoc comparisons further clarified that for many outcomes the two plyometric groups had greater performance post-intervention than the control. However, seated chest pass did not show between-group differences after adjustment. Accordingly, we interpret the present findings as the effect of adding a low-dose plyometric stimulus, and we acknowledge that the relatively low weekly frequency may partially explain why some between-group differences were less pronounced than might be expected under higher-frequency protocols.

For the overhead medicine ball throw outcome, the large improvement across time and significant interactions with both maturity and group reinforce the efficacy of plyometric stimulus on upper‐body ballistic power. Prior research in youth athletes has shown that upper-limb plyometric or combined upper/lower limb plyometric training can improve throwing measures (Hammami et al., 2020). In the context of maturation, systematic review evidence indicates that maturity can influence responsiveness to explosive training, although patterns may differ by outcome and the nature of the stimulus (Radnor et al., 2018). Importantly, the present greater improvements in more mature athletes interpretation should be considered alongside a potential ceiling (or scaling) effect. In many youth training contexts, less mature/pre-PHV athletes can show larger relative improvements because they start from lower baselines and may benefit disproportionately from motor learning and neural coordination changes, whereas post-PHV improvements can be supported more by morphological changes (Falk and Eliakim, 2003; Dotan et al., 2012). In our study, the maturity–response association was positive on absolute performance measures, suggesting that any ceiling constraints in post-PHV athletes did not dominate the observed changes, however, future analyses should consider presenting both absolute and relative (%Δ) changes and/or allometric scaling to disentangle baseline effects from true maturity-dependent trainability. Therefore, rather than attributing the maturity interaction solely to readiness, it is more appropriate to interpret it as the combined influence of maturity-related changes in size, coordination, and strength expression capacity, together with baseline-dependent room for improvement (Radnor et al., 2018).

Turning to the seated medicine ball chest pass, the time effect was very large, and significant time and maturity and time and group interactions were observed. Nonetheless, *post hoc* comparisons did not reveal significant between‐group differences after adjustment for multiple comparisons. This suggests that while all participants improved meaningfully, the three groups did not diverge significantly in their adjusted means. Literature investigating upper‐body plyometric effects in youth is somewhat limited, but combined upper/lower limb plyometric programs have shown improvements in medicine ball throw and other upper‐body power tests in adolescents  (Hammami et al., 2020). The absence of significant between‐group differences in our study might reflect that all groups (including control) improved, possibly through maturation or other training exposures, or that the chest-pass measure was less sensitive to distinguishing between protocols. Improvement in seated chest pass likely reflects enhancements in rate of force development, upper‐body sequential activation, and power transfer through the trunk–shoulder–arm chain, which are positively influenced by plyometric training and likely by maturation (e.g., increased muscle mass, improved motor unit recruitment) (Ramirez-Campillo et al., 2023). However, possible explanations (e.g., increased neural drive, tendon stiffness, or rate of force development) must be interpreted as plausible but unconfirmed in the present study, because we did not directly quantify neuromuscular activation, tendon/aponeurosis mechanical properties, muscle architecture, or laboratory-based rate of force development outcomes.

Regarding push-up height, our results showed a large time effect, a time and maturity interaction and a time and group interaction. Post hoc tests showed that at post-test PLYOgen exceeded control, whereas PLYObad did not reach significance relative to control. This suggests that the PLYOgen protocol may have been more effective for improving maximal push-up height in this study. The existing literature on upper-body plyometric training in youth is scarce, but the underlying mechanism is coherent with research showing that plyometric training improves upper-limb power via enhanced neuromuscular activation and stretch-shortening cycle efficiency  (Narang et al., 2022). Moreover, the maturity interaction suggests that more mature participants had slightly greater adaptation, consistent with findings that maturity status influences training responsiveness (Romero et al., 2021). At the same time, the contrasting equipment constraints of the two programmes imply that they likely targeted different regions of the force–velocity spectrum since PLYOgen (2-kg medicine-ball throws and explosive push-ups) represents a comparatively “overload” stimulus that emphasizes force/impulse production against an external mass, whereas PLYObad uses the very light badminton racket and thus more closely resembles a “velocity/overspeed” skill-constrained stimulus emphasizing rapid movement execution and coordination at high contraction velocities. This distinction provides a parsimonious explanation for why a generic overload-oriented programme could yield a clearer advantage in a generic ballistic task (push-up height), while both modalities may still transfer to smash speed via different proximal mechanisms (force capacity vs. velocity-specific coordination) (Hicks et al., 2023).

The badminton smash speed outcome exhibited very strong effects. Post-hoc analyses showed both plyometric groups were significantly better than control at post-test and irrespective of maturation. This finding suggests the transferability of plyometric training to sport-specific performance outcomes in badminton. These findings aligns with a systematic review indicating that plyometric and resistance/power training improve power, speed and racket-sport performance measures in youth athletes  (Deng et al., 2023). Improvements in smash speed likely result from enhanced upper-limb and trunk power, improved inter-segmental coordination, increased neural recruitment or improved rate of force development (Davies et al., 2015), all of which are known to respond to plyometric training and are enhanced with maturation (Radnor et al., 2018). To align interpretation with our measurement design, these mechanisms should be viewed as hypothesized pathways rather than mediators, given the absence of direct measures of activation, tendon stiffness, or segmental biomechanics (Dotan et al., 2012). In addition, although smash speed is a relevant and discriminative indicator of attacking capability, badminton performance is multidimensional since stroke accuracy/placement, decision-making under time pressure, fatigue resistance across rallies, and match outcomes also contribute materially to competitive success (Le Mansec et al., 2020; Sheng et al., 2025).

Despite these strengths, some limitations of the present study warrant mention. The study employed an additive design (intervention = regular badminton training + plyometrics; control = regular badminton training only), which confounds training content with total training volume. Accordingly, the present findings should be interpreted primarily as the effect of adding a weekly plyometric dose to usual practice, rather than definitive evidence that one plyometric modality is superior under volume-matched conditions. Future studies should include an active, volume-matched control (e.g., technical drills, mobility, or low-intensity skill work of equal duration) to better isolate the specific contribution of plyometric content. Moreover, our maturity assessment relied on maturity offset estimation, which is an indirect proxy for biological maturation and is subject to prediction error and potential systematic bias at the individual level. Therefore, moderation findings should be interpreted cautiously. Future work should consider complementary maturation indicators to reduce misclassification risk. Furthermore, although the intervention prescribed a standardized number of actions, we did not systematically monitor internal/external intensity, limiting inference on dose–response and the extent to which between-group differences reflect differences in effort or exposure quality. Matching programmes on the number of explosive actions does not ensure equivalence of mechanical dose (for instance impulse, peak force, rate of force development), eccentric demand, joint loading, or accumulated neuromuscular fatigue/parameters that may differ substantially between medicine-ball throws/explosive push-ups and jump-smash–based drills that incorporate approach steps, take-off, landing, and whole-body braking. As a result, some of the observed differences between PLYOgen and PLYObad could reflect unquantified differences in mechanical and physiological load rather than exercise specificity alone. Future studies should quantify session dose using complementary monitoring to improve dose matching. The sample size, while adequate for repeated-measures analyses, remains modest, and replication with larger and mixed-sex samples would enhance generalisability. Finally, the absence of detailed reliability quantification for all performance measures within the present sample may contribute to measurement error, and future work should report test–retest reliability and typical error to contextualize practical significance.

From a practical application, the findings support the inclusion of upper-body plyometric training within youth athletic development programs. Coaches should recognise that biological maturity moderates the magnitude of adaptation, and may therefore consider adjusting training progression, volume and intensity accordingly. Moreover, for badminton-specific tasks such as overhead throws or smash speed, the incorporation of plyometric drills appears beneficial beyond simply general resistance training. In planning youth training programmes, practitioners should monitor maturity offset and fit training expectations and progression to athlete’s needs. Practically, coaches may also consider combining overload-oriented exercises (as medicine-ball throws) with velocity/skill-specific overspeed elements (as rapid jump-smash variations) to target different regions of the force–velocity continuum while preserving technical quality and accuracy.

## 5 Conclusion

This study indicates that both upper-limb plyometric training and badminton-specific technical plyometrics can support improvements in selected upper-body explosive performance proxies (overhead medicine ball throw, seated chest pass, plyometric push-up height) and maximal smash speed in male youth badminton players over a 6-week period, although the magnitude of change varied across outcomes and was influenced by biological maturation. Compared with regular training alone, adding one supervised plyometric session per week was associated with greater improvements in overhead throw and smash speed, whereas between-intervention differences (PLYOgen vs. PLYObad) were outcome-dependent. Maturation-related effects should be interpreted cautiously because biological maturation was operationalized using a maturity-offset proxy rather than direct maturation assessment. These findings suggest that once-weekly structured plyometric training may be a useful complement to technical practice in young athletes, particularly when progression is individualized according to maturity status. Finally, appropriately progressed plyometric loading may plausibly enhance tissue capacity and movement robustness, thereby helping to mitigate injury risk while supporting appropriately adjusted load accommodation.

## Data Availability

The raw data supporting the conclusions of this article will be made available by the authors, without undue reservation.
